# Epigenetic orchestration by DNMT3B and DNMT3L throughout oocyte maturation

**DOI:** 10.1007/s00418-026-02486-6

**Published:** 2026-05-25

**Authors:** Nazlican Bozdemir, Ozgur Cinar, Fatma Uysal Cinar

**Affiliations:** 1 Department of Histology and Embryology, Ankara Medipol University School of Medicine, Altindag, 06050 Ankara, Turkey; 2 Department of Histology and Embryology, Ankara University School of Medicine, Altindag, 06080 Ankara, Turkey

**Keywords:** DNMT3B, DNMT3L, Epigenetic, Global DNA methylation, Oocyte maturation

## Abstract

DNA methylation represents a fundamental epigenetic mechanism regulating gene expression and developmental processes. In this study, we investigated the specific roles of DNMT3B and DNMT3L during mouse oocyte maturation, examined their reciprocal regulatory interactions, and assessed the potential compensatory responses of other DNA methyltransferases, including DNMT1 and DNMT3A, upon gene silencing. In addition, global DNA methylation levels were evaluated to elucidate the overall epigenetic impact of DNMT3B and DNMT3L silencing. In the study, siRNA specific to DNMT3B or DNMT3L was applied to germinal vesicle (GV) stage oocytes, which were then cultured under in vitro conditions. At the end of the culture period, metaphase II (MII) oocytes were collected, and DNMT protein expression levels and global DNA methylation status were analyzed using immunofluorescence staining specific to DNMT proteins and 5-methylcytosine (5mC), respectively. The results showed that silencing DNMT3B led to a significant decrease in DNMT3L protein levels, and vice versa (*p* < 0.001). DNMT3L silencing resulted in a significant increase in DNMT1 expression (*p* < 0.001), while DNMT3B silencing caused no significant change in DNMT1 levels. Both gene silencing conditions led to a significant reduction in DNMT3A expression (*p* < 0.001) and global DNA methylation levels (*p* < 0.001). Oocyte maturation was assessed by the proportion of GV-stage oocytes reaching the MII stage. A decreased maturation rate was observed in both DNMT3B and DNMT3L siRNA-treated groups. These findings indicate that DNMT3B and DNMT3L regulate each other and other DNMT enzymes, and play a key role in oocyte maturation by controlling DNA methylation dynamics.

## Introduction

Infertility remains a major global health concern, with a significant proportion of patients exhibiting abnormalities in oocyte maturation. Reports indicate that approximately 8.6–15.2% of infertile women produce at least one oocyte that fails to complete meiotic division (Bar-Ami et al. 1994; Avrech et al. 1997). When over 25% of the collected oocytes fail to reach full maturation, fertilization rates markedly decline, thereby reducing the probability of achieving a successful pregnancy (Bar-Ami et al. 1994). In clinical assisted reproductive technologies (ART), oocytes arrested at various stages of meiosis, namely germinal vesicle (GV, prophase I), metaphase I (MI), and metaphase II (MII), can often be recovered from the same individual, underscoring the heterogeneity in oocyte developmental competence (Chen et al. 2016).

Oocyte maturation is a complex, tightly regulated biological process governed by both genetic and epigenetic mechanisms. Among the latter, DNA methylation plays a central role in the regulation of gene expression during gametogenesis and early embryogenesis. This epigenetic modification involves the covalent addition of a methyl group to the 5th carbon of cytosine residues, primarily within CpG dinucleotides (Fraser and Lin 2016). DNA methylation is essential not only for the repression or activation of key developmental genes, but also for critical processes such as genomic imprinting and X chromosome inactivation (Leeke et al. 2025). Moreover, aberrant DNA methylation patterns have been associated with disrupted cell differentiation, tumorigenesis, aging, and impaired reproductive outcomes (Murase et al. 2024).

The enzymatic machinery responsible for establishing and maintaining DNA methylation includes the DNA methyltransferases (DNMTs), of which six isoforms have been identified in mammals: DNMT1, DNMT2, DNMT3A, DNMT3B, DNMT3C, and DNMT3L (Tóth et al. 2025). These enzymes function either in the maintenance of existing methylation marks (primarily by DNMT1) or in the establishment of new methylation patterns (mediated by DNMT3A and DNMT3B). Although DNMT3L lacks intrinsic catalytic activity, it enhances the de novo methylation efficiency of DNMT3A and DNMT3B by acting as an essential regulatory cofactor (Turek-Plewa and Jagodziński 2005).

Emerging evidence suggests that misregulation of DNMT gene expression may result in aberrant methylation landscapes, contributing to impaired oocyte maturation and infertility. For example, silencing of DNMT1 in ovine oocytes has been shown to arrest embryo development at the morula stage following in vitro fertilization, indicating the critical requirement of DNMT1 for blastocyst formation (Taylor et al. 2009). Similarly, in our previous study, knockdown of DNMT1 and DNMT3A has been associated with a significant reduction in murine oocyte maturation rates, alongside a concomitant decrease in DNMT3B and DNMT3L protein levels (Uysal et al. 2024). These findings raise the possibility that diminished expression of DNMT3B and DNMT3L may itself exert deleterious effects on meiotic progression and developmental competence. Accordingly, this study seeks to elucidate the critical functions of DNMT3B and DNMT3L in governing oocyte maturation and epigenetic regulation. It aims to define the specific contributions of these proteins to meiotic progression and developmental competence in mouse oocytes; to delineate the impact of DNMT3B and DNMT3L silencing on global DNA methylation landscapes; to characterize the compensatory regulatory dynamics among DNMT1, DNMT3A, and DNMT3L in response to DNMT3B suppression; and to investigate the compensatory responses of DNMT1, DNMT3A, and DNMT3B following targeted silencing of DNMT3L. Through these objectives, the study intends to provide mechanistic insight into the interplay between de novo DNA methyltransferases and the maintenance of oocyte epigenetic integrity.

In summary, our findings reveal that the silencing of DNMT3B and DNMT3L results in a decreased oocyte maturation rate, with a substantial proportion of oocytes arrested at the GV and MI stages. This disruption is accompanied by a significant reduction in global DNA methylation levels and a concomitant downregulation of DNMT3A expression in both knockdown groups. Notably, DNMT3L silencing induces a compensatory upregulation of DNMT1, whereas DNMT3B silencing does not elicit a similar response. Furthermore, a reciprocal regulatory relationship was observed between DNMT3B and DNMT3L, suggesting mutual dependence in maintaining proper methyltransferase expression. Collectively, these results highlight the indispensable and interdependent roles of DNMT3B and DNMT3L in supporting epigenetic homeostasis and ensuring the meiotic competence of mouse oocytes.

## Materials and methods

### Animals

The experimental protocol was approved by the Animal Care and Usage Committee of Ankara University (protocol no: 2024—16—138). The female Balb/C mice (*n* = 75) at 4–5 weeks of age were purchased from the Research Animal Laboratory Unit of Ankara University. All mice were hosted with free access to food and water and kept in a 12 h light/dark cycle.

### Collection of oocytes and in vitro culture

**Fig. 1:**
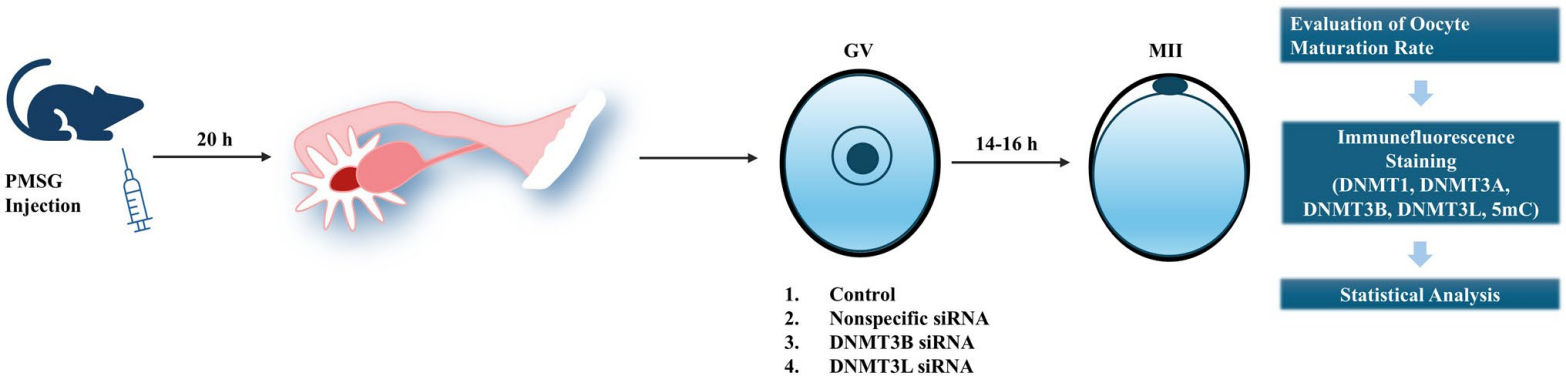
Experimental design. Following PMSG injection, GV-stage oocytes were collected from the ovaries and divided into control and experimental groups. Subsequently, MII-stage oocytes were retrieved, and oocyte maturation rates were evaluated. Immunofluorescence staining was performed, and global DNA methylation levels as well as protein expression levels were analyzed

Cumulus-enclosed germinal vesicle (GV)-stage oocytes were collected from the ovaries of 4-week-old Balb/C female micethat were superovulated with 5 IU pregnant mare’s serum gonadotropin (PMSG, Intervet, Milton Keynes, UK) and sacrificed through cervical dislocation. For this purpose, after removing adipose tissue surrounding the ovaries, cumulus-enclosed oocytes complexes (COC) containing fully grown GV-stage oocytes encircled by cumulus cells were released by puncturing the ovaries with a 23-gauge needle in morpholinepropanesulfonic acid (MOPS)-buffered medium (G-MOPS^™^) (Vitrolife, Göteborg, Sweden) (Fig. 1). The attached cumulus cells were removed by repetitive pipetting of COCs. Denuded GV oocytes were transferred to the culture medium (G-TL™; Vitrolife, Göteborg, Sweden) as 50 μL volumes of culture drops in 35 mm culture dishes (Corning, Corning, NY, USA) that were overlaid by approximately 3 mL of Ovoil (10,029, Göteborg,Vitrolife). GV oocytes (0 h) were cultured up to metaphase II (MII) oocytes (14–16 h) at 37 °C in 5% CO2. The criteria used to identify MII oocytes was the presence of polar body between the zona pellucida and oocyte.

### siRNA treatment

Lipid-based transfection method was used for siRNA delivery. Oocytes were transferred into 50 μL-drops of culture medium (G-TL^™^; Vitrolife, Göteborg, Sweden) containing 50 nM small interfering RNA (siRNA) oligonucleotides against DNMT3B (ON-TARGET plus SMART pool siRNAs L-044164—00—0005, Dharmacon, Lafayette, CO, USA) and against DNMT3L (ON-TARGET plus SMART pool siRNAs L-063056—01—0005, Dharmacon, Lafayette, CO, USA) versus nontargeting control siRNA duplexes for 14–16 h using DharmaFECT (Dharmacon, Lafayette, CO, USA) as a transfection reagent. Nontargeting siRNA duplex served as the control for siRNA. Transfection medium preparations were performed according to DharmaFECT™ Transfection Reagents—siRNA transfection protocol (Dharmacon, Lafayette, CO, USA).

### Immunofluorescence (IF) staining

Following fixation of MII oocytes for 20 min in 4% paraformaldehyde (Sigma-Aldrich, St. Louis, MO, USA), a permeabilization step was performed using 1% Tween-20 (Sigma-Aldrich, St. Louis, MO, USA) for 15 min at room temperature (RT). Subsequently, blocking was carried out for 1 h using a blocking solution containing 20% normal goat serum (Vector Laboratories, Newark, CA, USA). Immunofluorescence (IF) was applied to detect the relative quantity and cellular distribution profiles of the DNMT1, DNMT3A, DNMT3B, and DNMT3L proteins, and the relative levels of 5-methylcytosine (5mC) for global DNA methylation in the MII stage oocytes. Briefly, oocytes were incubated overnight at +4 °C with rabbit polyclonal antibody against DNMT1 (Abcam, ab87654, Cambridge, UK; reactive with either oocyte-specific (DNMT1o) or somatic (DNMT1s) isoforms), rabbit monoclonal antibodies against DNMT3A (Abcam, ab188470, Cambridge, UK), DNMT3B (Cell Signaling, 48,488, Danvers, MA, USA), DNMT3L (Abcam, ab3493, Cambridge, UK), or 5mC (Cell Signaling, 28692S, Danvers, MA, USA). Antibody specificity was validated by the manufacturers, and validation data are provided in the technical datasheets supplied by Abcam and Cell Signaling. For 5mC staining, oocytes were incubated in 2 M HCl for 30 min. Subsequent to an initial triple wash using 1 × PBS containing 2% bovine serum albumin (BSA) for a duration of 10 min each (PBS-BSA; Sigma-Aldrich, St. Louis, MO, USA), oocytes were subjected to an incubation with anti-rabbit IgG Alexa 488 secondary antibody (Invitrogen, California, USA) for a span of 1 h at RT. This was succeeded by another round of triple washes, utilizing the 1 × PBS-BSA solution, for 10 min each. For the negative control group, the primary antibodies were omitted to assess nonspecific binding of secondary antibody. The staining procedures were executed within miniwell trays (Thermo Fisher Scientific, Waltham, MA, USA) placed in a humidified chamber. Multiple 4 μL droplet of a PBS-based mounting medium, each containing 1 μg/mL of Hoechst dye (Thermo Fisher Scientific, Waltham, MA, USA) for DNA labeling, were prepared onto glass-bottomed 35 mm petri dishes. These droplets were then covered with paraffin oil. The stained oocytes were carefully placed within these droplets. All fluorescently tagged oocytes were kept intact in terms of their three-dimensional (3D) spherical shape and then were examined and imaged using a Zeiss LSM-880 Airyscan^®^ system (Zeiss, Oberkochen, Germany). Oocytes were classified as MII-stage based on standard morphological criteria and maturation timing under in vitro culture conditions. For signal intensity measurement, fluorescence channels were separated as green for DNMTs and 5mC and blue for Hoechst, and images were converted into 32-bit format. Total signal intensities were measured through pixel-based method by Image J software (National Institutes of Health, Bethesda, MD, USA). Fluorescence intensity measurements were performed within the oocyte cytoplasm while excluding the zona pellucida to avoid potential background signals. All images were acquired using identical microscope settings, and negative controls without primary antibody were included to verify staining specificity. Negative control signal intensities were subtracted, then signal intensity values normalized to the control group, and the relative staining intensity levels of the DNMT proteins and global DNA methylation have been quantified. For each experimental group, at least three independent replicates were performed, and representative images were selected from multiple fields. Quantitative analyses were conducted on a minimum of 150 oocytes per group.

### Statistical analysis

Images obtained from the immunofluorescence analyses were quantified using ImageJ software (National Institutes of Health, Bethesda, MD, USA). Statistical analyses were performed using a computer-based program (IBM SPSS) employing one-way analysis of variance (ANOVA), followed by Tukey’s post hoc test to identify differences among groups. A *p*-value of < 0.05 was considered statistically significant.

## Results

### Validation of DNMT3B and DNMT3L knockdown in oocytes

**Fig. 2:**
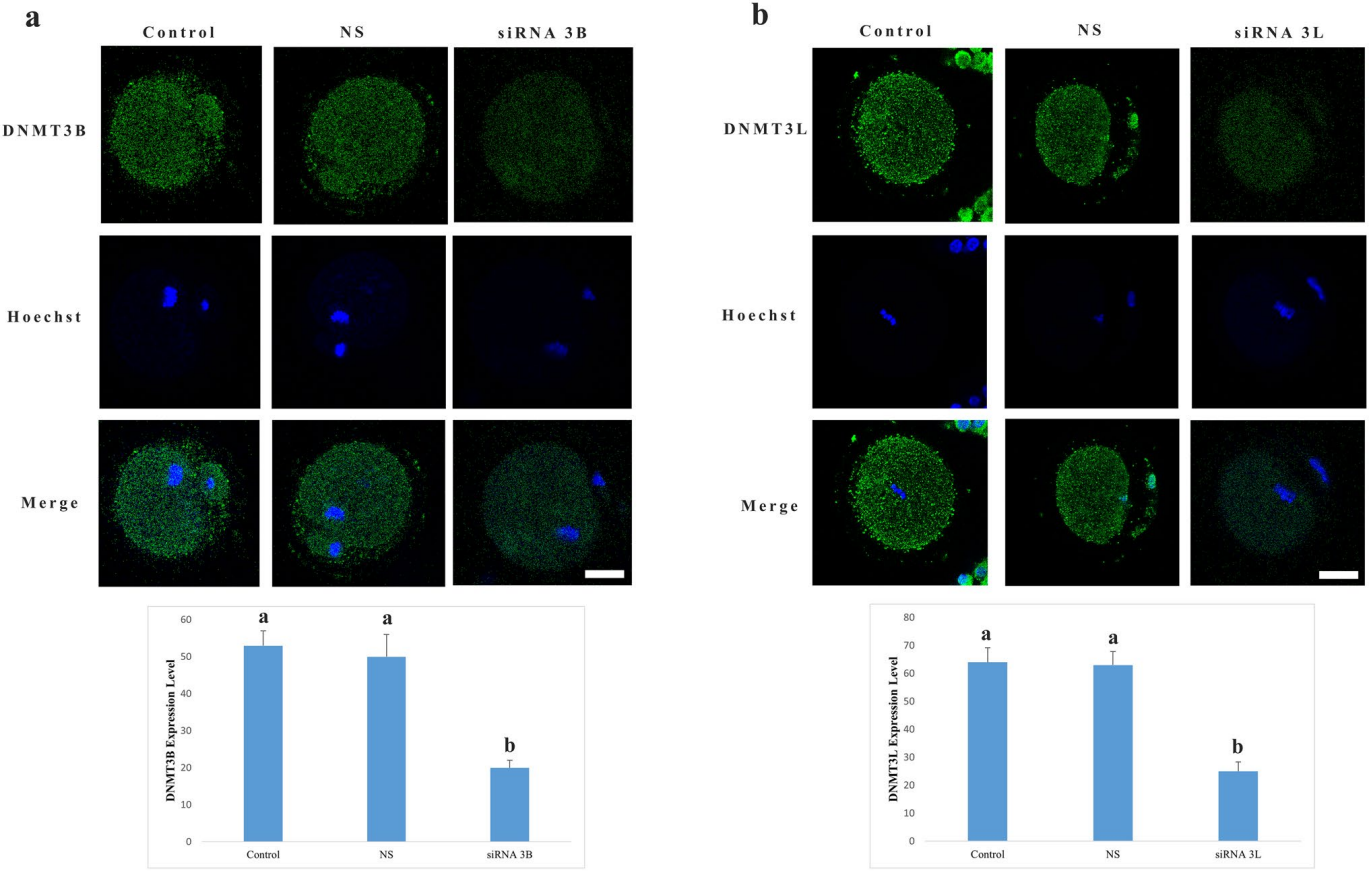
Silencing efficiency of DNMT3B and DNMT3L. **a** DNMT3B and **b** DNMT3L protein expression is visualized by green fluorescence; nuclei are counterstained with Hoechst (blue). Different letters in the graph indicate statistically significant differences between groups (*p* < 0.05). *NC* negative control group, *NS* nonspecific siRNA group. Scale bar = 20 µm

In oocytes treated with Dnmt3b-targeting siRNA, DNMT3B protein expression was significantly reduced compared with the control group (*p* < 0.001) (Fig. 2A). A similar and statistically significant decrease was also observed when compared with the group treated with nonspecific siRNA, indicating the specificity and efficacy of the silencing approach. In contrast, no significant difference in DNMT3B protein levels was detected between the control and nonspecific siRNA groups, both of which exhibited comparable levels of expression.

Likewise, in oocytes subjected to Dnmt3l siRNA treatment, DNMT3L protein levels were significantly decreased relative to both the control and nonspecific siRNA groups (*p* < 0.001) (Fig. 2B). Consistent with the findings for DNMT3B, DNMT3L expression did not differ significantly between the control and nonspecific siRNA groups, further supporting the specificity of the gene-targeted knockdown.

### DNMT3B expression level

**Fig. 3:**
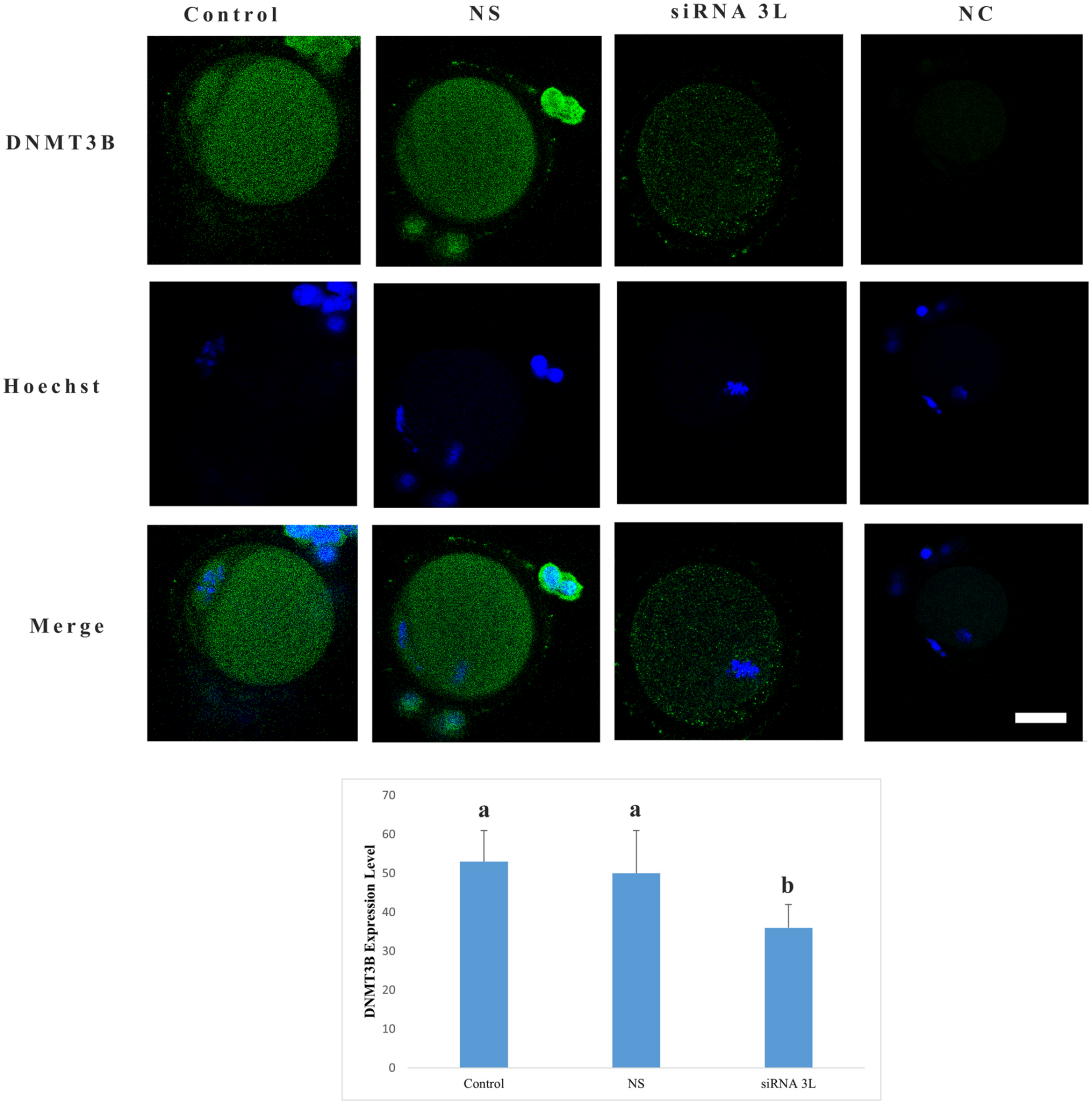
DNMT3B protein expression level. Expression of DNMT3B protein is visualized by green fluorescence, while nuclei are counterstained with Hoechst (blue). Different letters in the graph indicate statistically significant differences between groups (*p* < 0.05). *NC* negative control group, *NS* nonspecific siRNA group. Scale bar = 20 µm

Evaluation of DNMT3B protein localization demonstrated that in oocytes from the control group, DNMT3B showed finely granular, tiny grain-like distribution throughout cytoplasm (Fig. 3). In the DNMT3L siRNA-treated group, DNMT3B protein also exhibited finely granular staining; however, its expression level was significantly reduced compared with the control group (*p* < 0.001). In contrast, oocytes treated with nonspecific siRNA showed DNMT3B localization and signal intensity comparable to those of the control group, with no statistically significant difference observed (*p* = 0.921).

### DNMT3L expression level

**Fig. 4:**
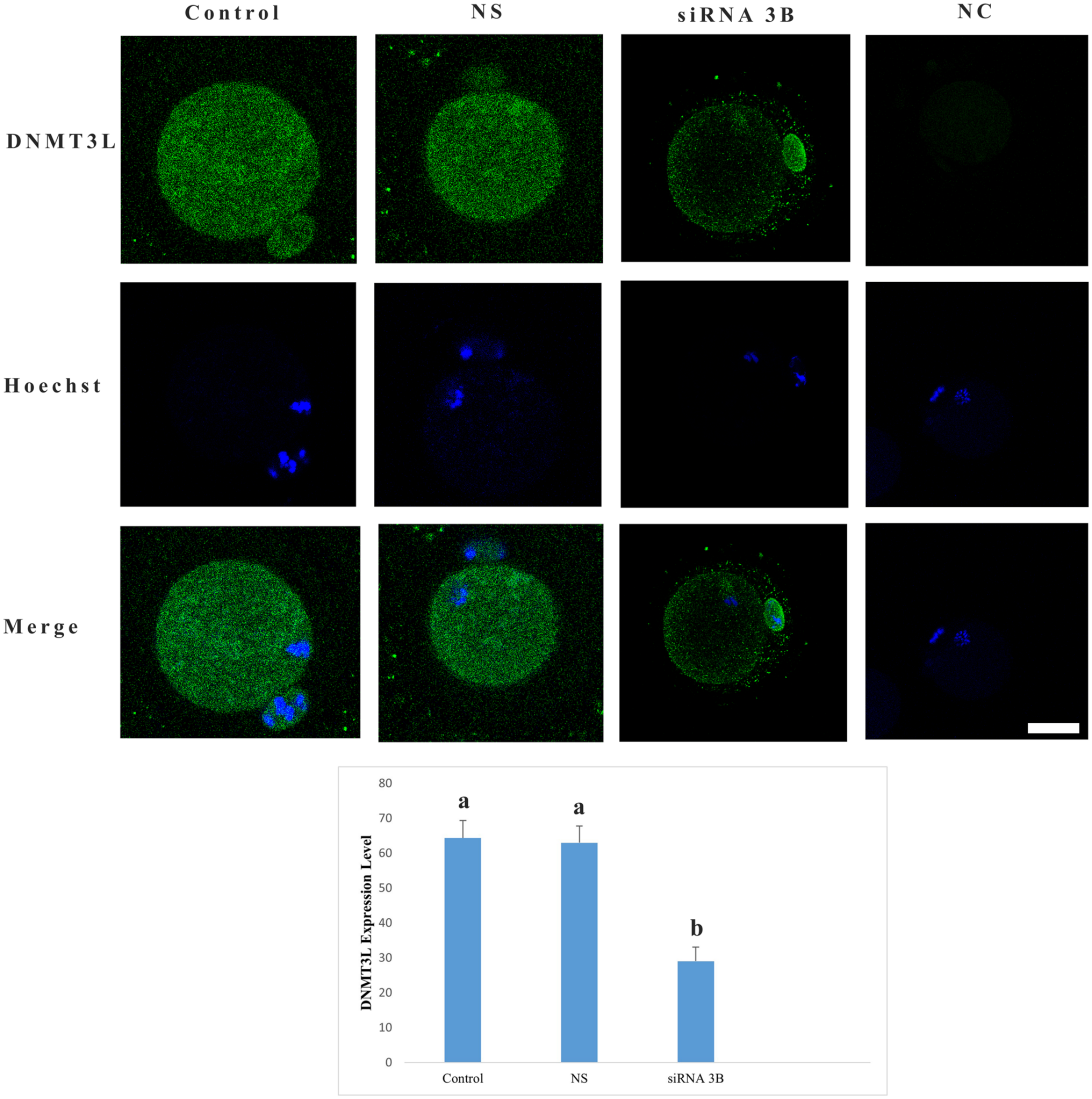
DNMT3L protein expression level. Expression of DNMT3L protein is visualized by green fluorescence, while nuclei are counterstained with Hoechst (blue). Different letters in the graph indicate statistically significant differences between groups (*p* < 0.05). *NC* negative control group, *NS* nonspecific siRNA group. Scale bar = 20 µm

In oocytes from the control group, DNMT3L protein demonstrated finely granular distribution throughout cytoplasm (Fig. 4). In the DNMT3B siRNA-treated group, the same distribution pattern was maintained; however, a statistically significant decrease in its expression level was observed compared with the control group (*p *< 0.001). In the group treated with nonspecific siRNA, both the intracellular localization pattern and signal intensity of DNMT3L were comparable to those of the control group, with no significant difference detected (*p* = 0.710).

### DNMT1 expression level

**Fig. 5:**
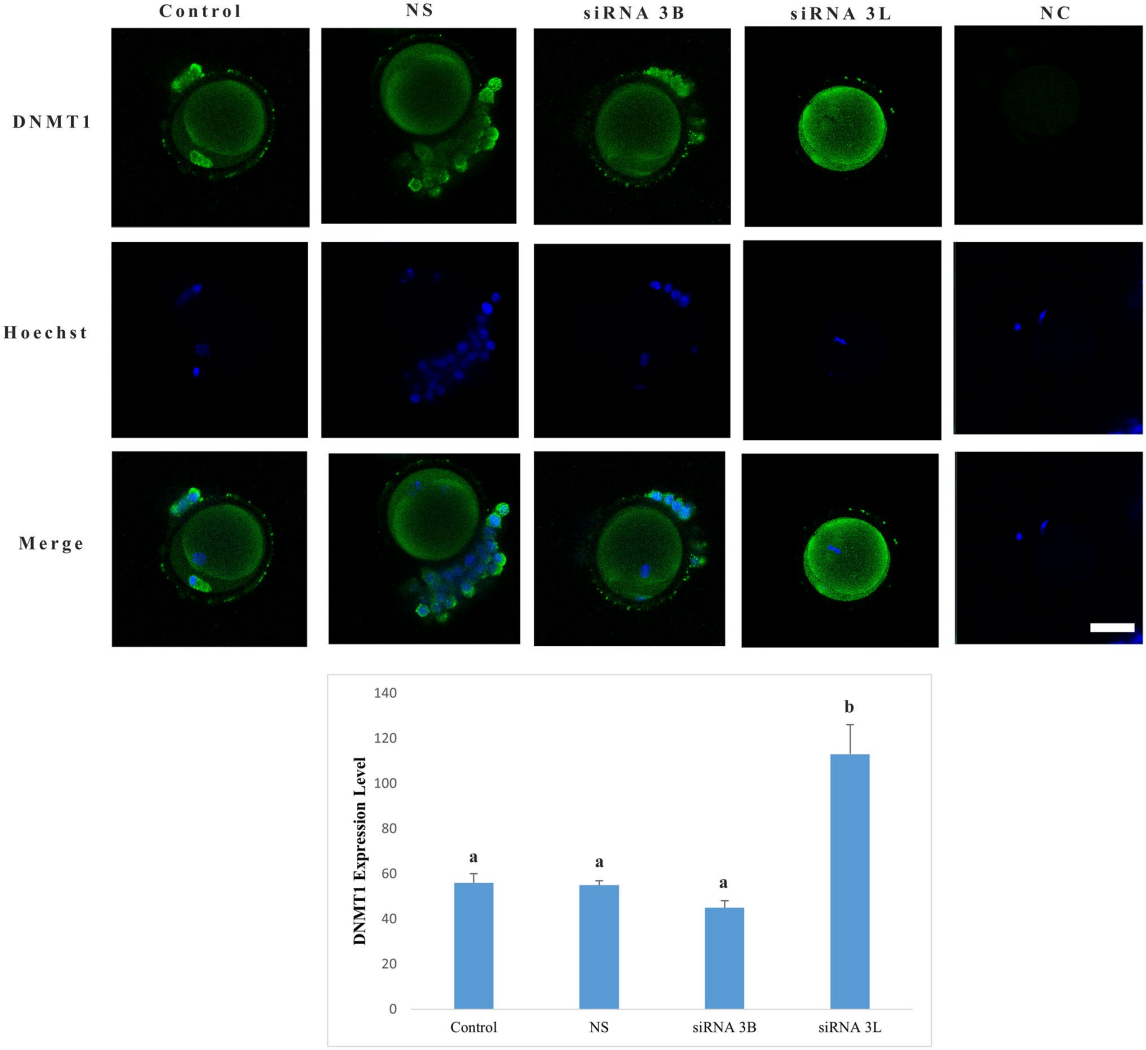
DNMT1 protein expression level. Expression of DNMT1 protein is visualized by green fluorescence, while nuclei are counterstained with Hoechst (blue). Different letters in the graph indicate statistically significant differences between groups (*p* < 0.05). *NC* negative control group, *NS* nonspecific siRNA group. Scale bar = 20 µm

DNMT1 protein exhibited throughout the cytoplasm across all experimental groups, with signal intensity notably concentrated in the subcortical region immediately beneath the oocyte membrane (Fig. 5). Quantitative analysis of expression levels revealed no significant differences in DNMT1 expression between the control, nonspecific siRNA (*p* = 0.997), and DNMT3B siRNA-treated groups (*p* = 0.115). In contrast, oocytes treated with DNMT3L siRNA displayed a statistically significant increase in DNMT1 protein expression compared with all other groups (*p* < 0.001).

### DNMT3A expression level

**Fig. 6:**
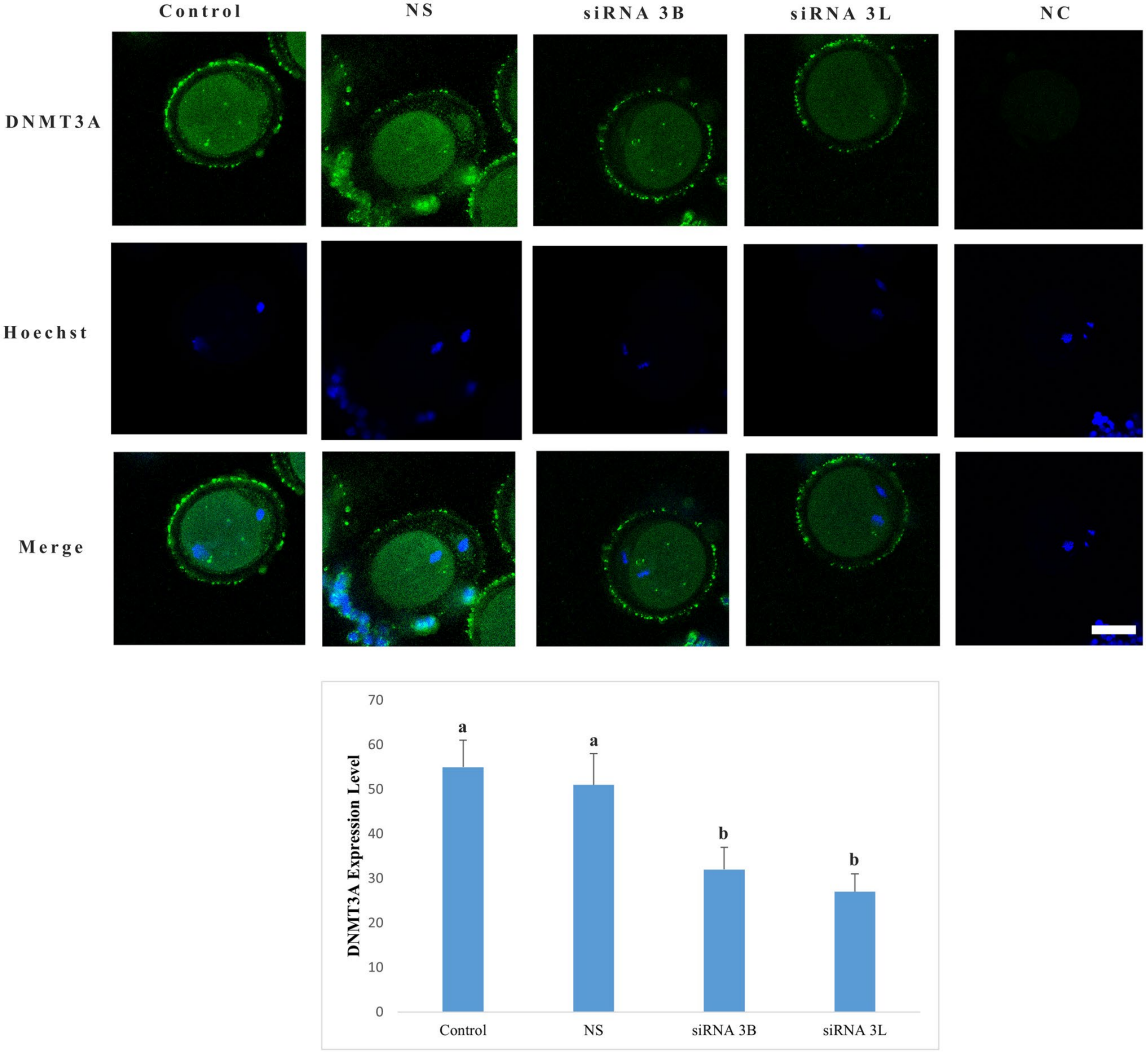
DNMT3A protein expression level. Expression of DNMT3A protein is visualized by green fluorescence, while nuclei are counterstained with Hoechst (blue). Different letters in the graph indicate statistically significant differences between groups (*p* < 0.05). *NC* negative control group, *NS* nonspecific siRNA group. Scale bar = 20 µm

DNMT3A protein was observed to be diffused throughout cytoplasm across all experimental groups (Fig. 6). Analysis of expression levels demonstrated no statistically significant difference between the control and nonspecific siRNA groups (*p* = 0.564). However, in both the DNMT3B and DNMT3L siRNA-treated groups, DNMT3A protein levels were significantly reduced compared with both control groups (*p* < 0.001). No statistically significant difference was detected between the DNMT3B and DNMT3L siRNA groups in terms of DNMT3A expression (*p* = 0.391).

### Global DNA methylation

**Fig. 7:**
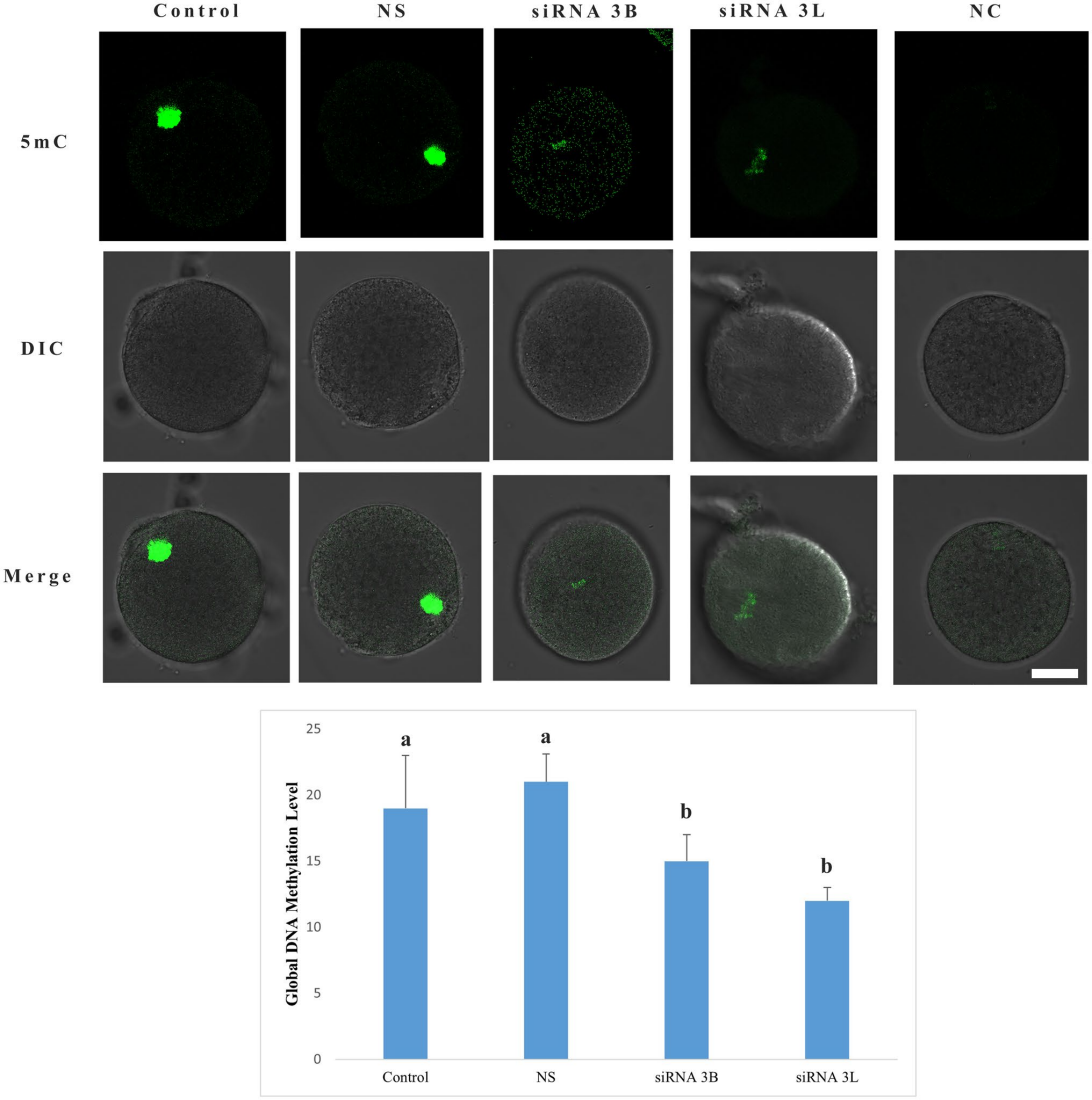
Global DNA methylation level. Global DNA methylation was detected using 5-methylcytosine (5mC) and visualized as a green fluorescence signal. Different letters in the graph indicate statistically significant differences between groups (*p* < 0.05). *DIC* differential interference contrast, *NS* nonspecific siRNA group, *NC* negative control group. Scale bar = 20 µm

To assess global DNA methylation levels, immunofluorescent staining for 5-methylcytosine (5mC) was performed. As expected, 5mC signals were concentrated on the chromosomes of oocytes at the metaphase II stage (Fig. 7). No statistically significant difference in signal intensity was observed between the control and nonspecific siRNA groups (*p* = 0.484). In contrast, both DNMT3B and DNMT3L siRNA-treated groups exhibited a significant reduction in global DNA methylation levels compared with controls (*p* < 0.001). However, no statistically significant difference was detected between the DNMT3B and DNMT3L siRNA groups (*p* = 0.076).

### Alterations in maturation dynamics following the silencing of DNMT3B and DNMT3L

**Fig. 8:**
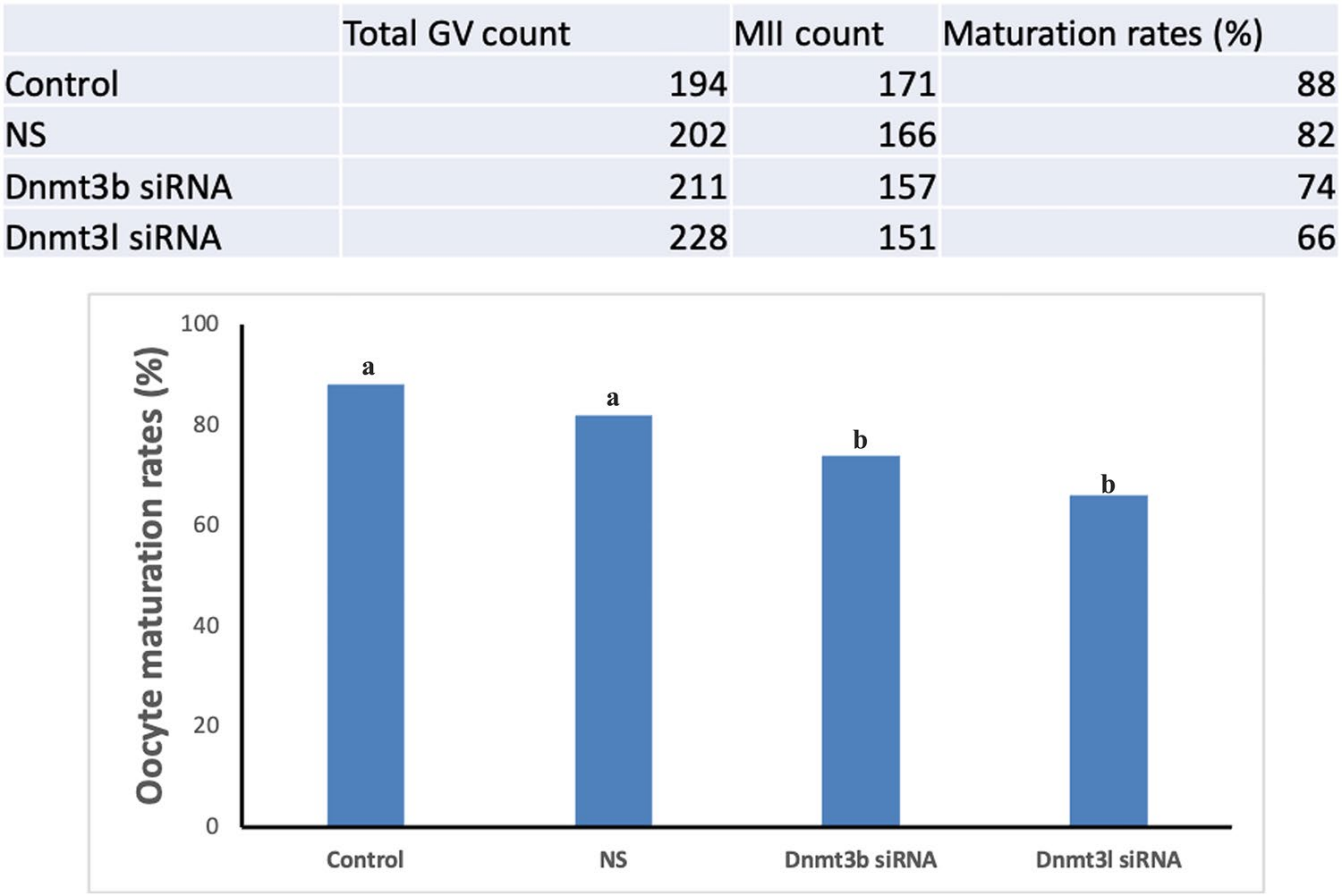
Effects of DNMT3B and DNMT3L knockdown on oocyte maturation. The table presents the total number of germinal vesicle (GV) stage oocytes collected, the number of oocytes reaching the MII stage, and the calculated maturation rates for each experimental group. The bar graph represents the percentage of oocytes that progressed to the MII stage after in vitro maturation. The nontargeting siRNA (NS) group did not differ from the control, whereas both Dnmt3b and Dnmt3l knockdown significantly reduced maturation rates. Different letters above bars indicate statistically significant differences between groups (*p* < 0.05)

In this study, the in vitro maturation rates of germinal vesicle (GV)-stage oocytes to the metaphase II (MII) stage were evaluated. GV-stage oocytes were cultured for 14–16 h under in vitro conditions (Bozdemir et al. 2025), after which they were examined under a stereomicroscope to determine the number that had progressed to the MII stage. A total of 194 control, 202 nontargeting siRNA, 211 DNMT3B siRNA, and 228 DNMT3L siRNA-treated oocytes were analyzed. The proportion of oocytes reaching the MII stage was 88% (171/194) in the control group and 82% (166/202) in the nontargeting siRNA group. In the DNMT3B and DNMT3L siRNA groups, maturation rates were 74% (157/211) and 66% (151/228), respectively. Statistical analysis based on the raw counts confirmed significant reductions in maturation rates in the DNMT3B and DNMT3L siRNA groups compared with control, whereas the nontargeting siRNA group did not differ significantly from control (Fig. 8).

## Discussion

**Fig. 9:**
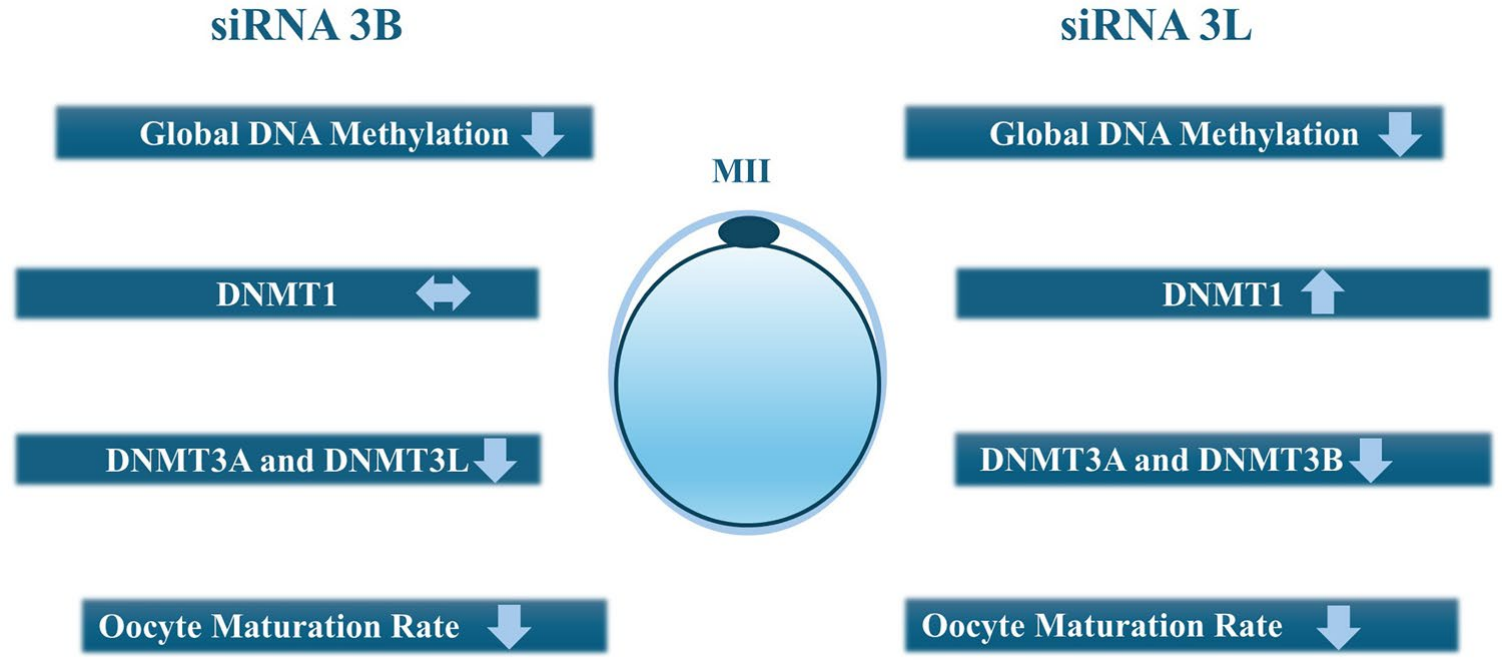
A schematic summary of the findings. This diagram illustrates the effects of DNMT3B and DNMT3L gene silencing on DNMT enzyme levels, global DNA methylation status, and oocyte maturation in metaphase II (MII)-stage oocytes

This study investigated the effects of DNMT3B and DNMT3L silencing via siRNA technology on protein expression levels, global DNA methylation patterns, and in vitro maturation success in mouse oocytes. Both DNMT3B and DNMT3L proteins exhibited finely granular distribution throughout the cytoplasm, consistent with previous findings from our laboratory in which siRNA-mediated silencing of DNMT1 and DNMT3A was shown to alter DNMT3B and DNMT3L expression in MII-stage oocytes (Uysal et al. 2024). The cytoplasmic localization observed at the MII stage is consistent with the absence of the nuclear envelope and with the known storage of maternal epigenetic regulators in mature oocytes. The cytoplasmic localization of DNMT3L, despite its lack of catalytic activity, further supports its structural role in guiding methylation processes through complex formation with DNMT3A/B (Jia et al. 2007). Interestingly, DNMT3L silencing resulted in a significant upregulation of DNMT1 protein levels (Fig. 9), suggesting the presence of a compensatory mechanism within the epigenetic network since it is shown that DNMT1 may exhibit limited de novo methyltransferase activity in the absence of DNMT3 proteins even though its primary function is maintenance in DNA methylation (Jurkowska et al. 2011; Feltus et al. 2003; Jair et al. 2006; Fatemi et al. 2002). Conversely, DNMT3B silencing did not alter DNMT1 expression, implying a lack of compensatory regulation. DNMT3B silencing resulted in downregulation of DNMT3L, consistent with a previous study demonstrating that DNMT3A and DNMT3B regulate DNMT3L expression through methylation of the *Dnmt3l* promoter, a process that involves DNMT3L itself (Hu et al. 2008).

The significant decrease in DNMT3A levels observed in both knockdown groups further supports the hypothesis of a tightly regulated molecular balance among DNMT3 family members. Previous studies have shown that these enzymes function cooperatively to establish and maintain methylation patterns, and that the deficiency of one may directly impact the expression or activity of the others (Gowher et al. 2005). Consistent with this, our findings show dramatic reduction in global DNA methylation following the silencing of either DNMT3B or DNMT3L, as demonstrated by 5-methylcytosine (5mC) immunostaining. These results reinforce the central role of DNMT3 family members in de novo methylation processes (Okano et al. 1999).

The observed reduction in maturation rates following DNMT3B and DNMT3L targeting suggests that even transient interference with de novo DNA methylation machinery during the maturation window may compromise oocyte developmental competence. Notably, the nontargeting siRNA group did not differ from the untreated control, indicating that the observed effects are unlikely to result from the transfection procedure itself.

Although the duration of siRNA exposure during in vitro maturation may not be sufficient to induce global methylation changes, the results suggest that DNMT activity during this critical window may contribute to the regulation of maturation-related processes, possibly through localized or gene-specific epigenetic modulation. The more pronounced reduction observed in the DNMT3L-targeted group is consistent with the known role of DNMT3L as a cofactor that stabilizes de novo methyltransferase complexes, suggesting that perturbation of this regulatory component may have broader functional consequences during oocyte maturation.The reduced progression from GV to MII stage is in line with our previous findings, where silencing of DNMT1 and DNMT3A similarly resulted in impaired maturation outcomes (Uysal et al. 2024), thereby reaffirming the critical involvement of DNMT family enzymes in oocyte maturation.

This study comprehensively investigated the epigenetic consequences of DNMT3B and DNMT3L silencing in mouse oocytes. The significant reduction in DNMT3 protein levels upon silencing of genes may indicate a molecular regulatory interconnection among DNMT3 family members, while upregulation of DNMT1 following DNMT3L knockdown may suggest a compensatory, potential feedback mechanisms.

Partial decrease in oocyte maturation following DNMT3B and DNMT3L depletion suggests that these factors contribute to, but are not solely essential for, meiotic progression. The ability of a considerable proportion of oocytes to reach the MII stage indicates the presence of compensatory or preexisting regulatory mechanisms. One possible explanation is the heterogeneity of GV-stage oocytes in terms of chromatin configuration, namely non-surrounded nucleolus (NSN) and surrounded nucleolus (SN) states, which differ in their transcriptional activity. Since NSN oocytes are transcriptionally active whereas SN oocytes are largely transcriptionally silent, the impact of siRNA-mediated depletion of DNMT3B and DNMT3L may vary depending on the transcriptional status at the time of treatment. In this context, SN oocytes may be less affected due to reduced reliance on ongoing transcription, whereas NSN oocytes could be more sensitive to the depletion of these epigenetic regulators. In addition, the persistence of DNMT3B and DNMT3L proteins synthesized prior to siRNA treatment may contribute to the maintenance of meiotic progression. Residual protein activity may partially sustain essential methylation-dependent processes required for oocyte maturation despite reduced transcript levels. Importantly, progression to the MII stage alone does not necessarily reflect functional competence. Epigenetic perturbations resulting from DNMT3B and DNMT3L depletion may impair oocyte quality, fertilization capacity, and subsequent embryonic development without markedly affecting morphological maturation. Therefore, maturation rates alone may underestimate the functional consequences of DNMT depletion.

Future studies could further expand upon the present findings. For instance, DNA methylation dynamics may be investigated with higher resolution using gene-specific or imprinting region analyses instead of global 5mC immunostaining. Protein expression could be quantified more precisely, and transcriptional alterations validated at the mRNA level. Advanced approaches such as bisulfite sequencing, MeDIP-seq, or genome-wide methylation profiling, combined with quantitative polymerase chain reaction (qPCR) and Western blot analyses, would provide deeper mechanistic insights. Moreover, Clustered Regularly Interspaced Short Palindromic Repeats (CRISPR)/dCas9-based epigenetic editing systems could enable locus-specific interrogation of DNMT functions.

In addition, future studies should address certain limitations of the present work. GV-stage oocytes were not classified according to their chromatin configuration (NSN versus SN), and the potential contribution of preexisting DNMT3B and DNMT3L protein pools was not evaluated. Functional outcomes such as fertilization capacity, early embryonic development, and implantation efficiency were also not assessed. Therefore, although meiotic maturation was preserved, potential effects on oocyte quality and developmental competence cannot be excluded.

Further investigations could examine how Dnmt3b and Dnmt3l knockdown affects in vitro fertilization (IVF) outcomes, early embryonic development, and implantation efficiency within the endometrium. The study was designed to assess whether transient perturbation of DNMT activity during the maturation window affects oocyte competence. Future studies including analysis of oocytes failing to reach MII may further clarify stage-specific effects. Finally, long-term studies evaluating the health, epigenetic landscape, and reproductive competence of offspring derived from DNMT-deficient oocytes would yield valuable information on the intergenerational impacts of altered oocyte epigenetics.

## Conclusions

This study highlights the indispensable and coordinated functions of DNMT3B and DNMT3L in regulating oocyte epigenetic integrity and developmental competence. These findings contribute to a deeper understanding of the epigenetic mechanisms underlying fertility and early embryogenesis, and provide a conceptual framework for the development of targeted epigenetic therapeutic strategies aimed at improving oocyte quality, maturation outcomes, and overall success rates in assisted reproductive technologies, particularly in the setting of in vitro fertilization (IVF).

## Data Availability

No datasets were generated or analyzed during the current study.
